# Public Health Laboratory Work

**Published:** 1915-06

**Authors:** 


**Public Health Laboratory Work.** By HENRY R. KENWOOD, M.B., F.R.S. Edin. Sixth Edition. Pp. xi., 418. London: H. K. Lewis. 1914. Price, 10s. net.

There is evidence of careful revision throughout in this edition of Professor Kenwood's well-known text-book. The scope of the book is now restricted to the clinical branch of Public Health laboratory work, and the former short résumé of bacteriological work is omitted, thus allowing the chemical work to be dealt with more completely without unduly increasing the size of the book. It is a safe and reliable guide for the Public Health student.

